# Preoperative Fasting C-Peptide Acts as a Promising Predictor of Improved Glucose Tolerance in Patients With Acromegaly After Transsphenoidal Surgery: A Retrospective Study of 64 Cases From a Large Pituitary Center in China

**DOI:** 10.3389/fendo.2019.00736

**Published:** 2019-11-01

**Authors:** Zihao Wang, Lu Gao, Xiaopeng Guo, Chenzhe Feng, Kan Deng, Wei Lian, Ming Feng, Xinjie Bao, Bing Xing

**Affiliations:** ^1^Department of Neurosurgery, Peking Union Medical College Hospital, Chinese Academy of Medical Sciences and Peking Union Medical College, Beijing, China; ^2^Chinese Pituitary Adenoma Cooperative Group, China Pituitary Disease Registry Center, Beijing, China

**Keywords:** acromegaly, C-peptide, glucose intolerance, diabetes mellitus, transsphenoidal surgery

## Abstract

**Purpose:** Abnormal glucose metabolism is one of the most frequent acromegaly complications. Improvement of glucose metabolism can be observed only in half of acromegaly patients after surgery. We aimed to investigate the risk factors for determining abnormal glucose metabolism before surgery in patients with acromegaly, and to explore the predictors of improved preoperative glucose intolerance after surgery.

**Methods:** We retrospectively reviewed 64 patients who received transsphenoidal surgery for acromegaly. Growth hormone (GH), insulin-like growth factor-1 (IGF-1) and glucose metabolism were assessed before, immediately after, and 3 months after surgery. Glucose metabolic parameters included glycosylated hemoglobin (HbA1c), plasma glucose (PG), C-peptide (CP), insulin (INS), and the indices of β-cell function, insulin sensitivity, and insulin resistance (IR).

**Results:** Preoperatively, 18 patients (28.1%) had diabetes (DM), 34 (53.1%) had prediabetes (PreDM), and 12 (18.8%) had normal glucose tolerance (NGT). All the indices of pancreatic β-cell function were significantly lower in patients with DM than those with PreDM and NGT (all *P* < 0.005). IGF-1 was significantly positively correlated with insulin sensitivity and IR (*P* < 0.05), while GH was not. Postoperatively, glucose tolerance was improved in 71.2% of patients (37/52) with preoperative glucose intolerance. Insulin sensitivity was increased, while β-cell function and IR were decreased in most patients after surgery, regardless of whether their acromegaly achieved remission. A multivariate logistic regression analysis revealed that preoperative fasting C-peptide (FCP, OR = 2.639, *P* = 0.022), disposition index (DI, OR = 1.397, *P* = 0.043) and Predictor-2 (OR = 0.578, *P* = 0.035) were determined to be the predictors for improved glucose tolerance status after surgery. Afterwards, through Receiver operating characteristic (ROC) analyses, FCP >2.445 ng/ml was the best independent predictor, with an 86.6% PPV (positive predictive value) and a 74.5% NPV (negative predictive value).

**Conclusions:** Preoperative high FCP is a promising postsurgical predictor of improved glucose tolerance in patients with acromegaly. Oral glucose tolerance testing (OGTT) and HbA1c should be monitored regularly after surgery, and diabetes management should be adjusted based on the patient's latest glucose tolerance status.

## Introduction

Growth hormone-secreting pituitary adenomas are characterized by excessive growth hormone (GH) and insulin-like growth factor-1 (IGF-1) secretion, which consequently results in a series of metabolic disorders (1). Glucose metabolism alterations, including diabetes mellitus (DM), and prediabetes (impaired fasting glucose [IFG], and/or impaired glucose tolerance [IGT]), are recognized as one of the most frequent acromegaly complications with prevalences ranging from 12 to 56% (2–7). Glucose intolerance further contributes to increased cardiovascular risk and mortality (5–7). Transsphenoidal adenectomy (TSA) is the first-line treatment for acromegaly (1). GH and IGF-1 levels decline rapidly and sharply after successful surgery, which normalizes the glucose metabolism in 23–58% of patients with preoperative diabetes per previous studies (2–8). Our clinical experience has shown that glucose metabolism improves in almost half of acromegaly patients with glucose intolerance. However, why some patients' glucose tolerance status fails to improve postoperatively and which factors are involved, such as GH, IGF-1, acromegaly remission status, pancreatic β-cell function, insulin sensitivity and insulin resistance (IR), remains unclear. Moreover, how to predict the surgical benefit to patients with abnormal glucose tolerance before surgery is also a concern for neurosurgeons and endocrinologists.

In our study, we investigated the risk factors for determining and predicting preoperative glucose intolerance in patients with acromegaly. We also explored the associated and predictive parameters of improved postoperative glucose metabolism in patients with glucose intolerance before surgery. Finally, because no internationally agreed upon guideline exists for managing impaired glucose metabolism in acromegaly, we hope our study provides new evidence for therapeutic strategies for glucose intolerance in acromegaly patients.

## Materials and Methods

### Patient Populations

We retrospectively analyzed consecutive patients diagnosed with GH-secreting pituitary adenomas at Peking Union Medical College Hospital (PUMCH) between January 2017 and September 2018. Acromegaly was diagnosed using the following criteria: (1) an elevated serum IGF-1 level, (2) a nadir serum GH ≥0.4 μg/L after an oral glucose load, (3) a positive magnetic resonance imaging (MRI) finding, and (4) acromegalic manifestations, including acral enlargement, DM, hypertension and sleep apnoea (9). Sixty-four newly diagnosed and untreated patients with acromegaly (35 men and 29 women) were enrolled without previous transsphenoidal surgery, radiotherapy or medical treatment preoperatively. Patients on insulin therapy were excluded due to the confounding effect of exogenous insulin administration. The mean age was 41.1 ± 11.0 years (ranging from 17 to 70 years). The mean symptom duration was 6.3 ± 4.4 years. The average follow-up duration was 3 months. All 64 patients underwent similar microscopic transsphenoidal adenectomy performed by experienced neurosurgeons. Per the current clinical practice guidelines released in 2014, patients were divided into 3 categories after surgery: (1) the remission group: normalized GH, including random GH <1.0 μg/L or nadir GH <0.4 μg/L after oral glucose tolerance testing (OGTT) and normalized age- and sex-adjusted IGF-1; (2) the GH/IGF-1 discordant group: normalized GH and elevated IGF-1 or elevated GH and normalized IGF-1; and (3) the non-remission group: elevated GH and elevated IGF-1 (9).

Glucose tolerance was evaluated using the glucose criteria of either the fasting plasma glucose (FPG) or the 75-g OGTT. Per the 2019 American Diabetes Association (ADA) practice guidelines for diabetes, DM was diagnosed when the FPG was ≥7.0 mmol/L or the 2-h plasma glucose (2h-PG) was ≥11.1 mmol/L, or the glycosylated hemoglobin (HbA1c) was ≥6.5%. Prediabetes (PreDM) includes IFG (FPG 5.6–6.9 mmol/L), and/or IGT (2h-PG 7.8–11.0 mmol/L), and/or HbA1c 5.7–6.4%. Normal glucose tolerance (NGT) was diagnosed when the FPG was <5.6 mmol/L and the 2h-PG was <7.8 mmol/L (10). Fourteen patients with histories of glucose intolerance before surgery were treated with oral hypoglycaemic agents (metformin, acarbose, or insulin secretagogues). To ensure OGTT assessment accuracy, oral hypoglycaemic agents were temporarily stopped for at least 12 h, and insulin secretagogues (sulfonylurea and nateglinide) were stopped for at least 2–3 days prior to OGTT (10). Postoperatively, patients were divided into 3 categories based on their glucose tolerance status change before and after surgery: (1) the improved group: either from DM to PreDM or NGT or from PreDM to NGT; (2) the unimproved group: from DM to DM, PreDM to PreDM, or NGT to NGT; and (3) the deteriorative group: either from PreDM to DM or from NGT to PreDM or DM.

All procedures involving human participants were performed in accordance with the ethical standards of the Institutional Ethics Committee of Peking Union Medical College Hospital at the Chinese Academy of Medical Sciences & Peking Union Medical College and with the 1964 Declaration of Helsinki and its later amendments or comparable ethical standards. Informed consent was obtained from all participants included in the study.

### Biochemical Assessments

All patients' endocrine and glucose metabolic parameters were assessed before, immediately after, and 3 months after surgery.

Endocrine parameters included GH and IGF-1. Serum GH levels were measured via immunoradiometric assays. Random GH was measured in the fasting condition without glucose loading. A 75-g OGTT was performed after overnight fasting. Serum GH levels were evaluated at 0, 30, 60, 120, and 180 min after orally administering 75 g of glucose. The nadir GH was defined as the lowest GH value measured via the OGTT. Serum IGF-1 was measured without glucose loading using immunochemiluminescence assays. IGF-1 was expressed as the age- and sex-adjusted standardized forms (IGF-1 [%ULN]), which is the percentage of the upper limit of normal (ULN) based on data from the healthy Chinese population obtained from the PUMCH Department of Laboratory Medicine (11, 12).

Glucose metabolic parameters included HbA1c, plasma glucose (PG), C-peptide (CP), insulin (INS), and the β-cell function indices, insulin sensitivity and IR. HbA1c was measured via high-performance liquid chromatography. PG was measured using the hexokinase method. INS and CP were measured using chemiluminescence immunoassays. PG, CP and INS levels were evaluated at 0, 30, 60, 120, and 180 min after the 75-g OGTT. Pancreatic islet β-cell functioning was evaluated from the INS; CP; the homeostasis assessment models of β-cell function (HOMA1-%β [INS]) (13, 14), HOMA2-%β (INS) (15), and HOMA2-%β (CP) (15); the areas under the curve for INS (AUC_INS_) (16), AUC_CP_, AUC_INS_/AUC_PG_ (17), and AUC_CP_/AUC_PG_; the insulinogenic index (IGI) (18); IGI/IR; the disposition index (DI) (19); the OGTT insulin secretion sensitivity index 2 (ISSI2) (20); the modified β-cell function index (MBCI) (21); estimated first-phase insulin release (eFPIS) and estimated second-phase insulin release (eSPIS) (22). Insulin sensitivity was evaluated using the homeostasis assessment models of insulin sensitivity (HOMA1-%S [INS]) (13, 14), HOMA2-%S (INS) (15), HOMA2-%S (CP) (15); the quantitative insulin sensitivity check index (QUICKI) (23); the Matsuda index (whole-body insulin sensitivity index, WBISI) (24, 25) and the estimated metabolic clearance rate of glucose (eMCR) (22). Insulin resistance was evaluated by the homeostasis assessment models of insulin resistance (HOMA1-IR [INS]) (13, 14), HOMA2-IR (INS) (15), and HOMA2-IR (CP) (15), and the insulin activity index (IAI) (25). Supplementary Table 1 lists the calculation formulas. Notably, we used both INS and CP to calculate HOMA parameters to evaluate the glucose metabolism of acromegalic patients. Because CP is commonly used to evaluate pancreatic β-cell functions in diabetic patients, while CP is slightly less accurate than insulin when evaluating IR. So we believe INS and CP has their advantages and disadvantages in evaluating glucose metabolism.

### Statistical Analysis

Statistical analyses were performed using SPSS 22.0 (SPSS, Inc., Chicago, Illinois, USA). Normally distributed continuous variables are expressed as the means ± standard deviations, and abnormally distributed continuous variables are expressed as the medians (interquartile ranges). Categorical variables are expressed as numbers (percentages). The independent Student's *t*-test for continuous data and the χ^2^ test for categorical data were used to compare two groups. One-way analysis of variance (ANOVA) and the Kruskal-Wallis test were used to compare multiple groups. Correlations between normally distributed variables were assessed using Pearson's correlation test, while abnormal distributions were assessed using Spearman's rho test. Logistic regression analysis was used to assess the risk factors for glucose intolerance before surgery and assess the parameters for predicting an improved glucose tolerance status after surgery. The predictor was the predicted value calculated by the prediction model using logistic regression. Receiver operating characteristic (ROC) analyses were performed to investigate the predictive value of these parameters, including predictors from the logistic regression analysis. Areas under the curve (AUCs), optimal cut-off values, sensitivity, and specificity were calculated. *P*-values < 0.05 were considered statistically significant. Confidence intervals (CIs) were set at 95%.

## Results

### Preoperative Glucose Tolerance Status and Glucose Metabolic Parameters

**Figure 1:**
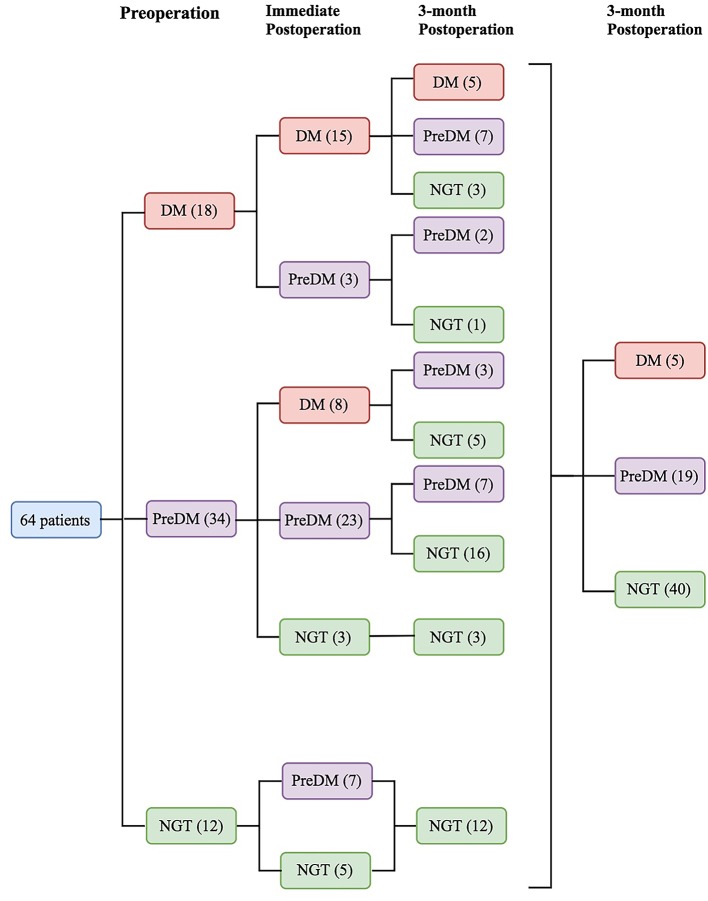
Sixty-four patients were divided into 3 categories based on glucose tolerance status before and after surgery: diabetes mellitus (DM), prediabetes (PreDM), or normal glucose tolerance (NGT).

**Figure 2:**
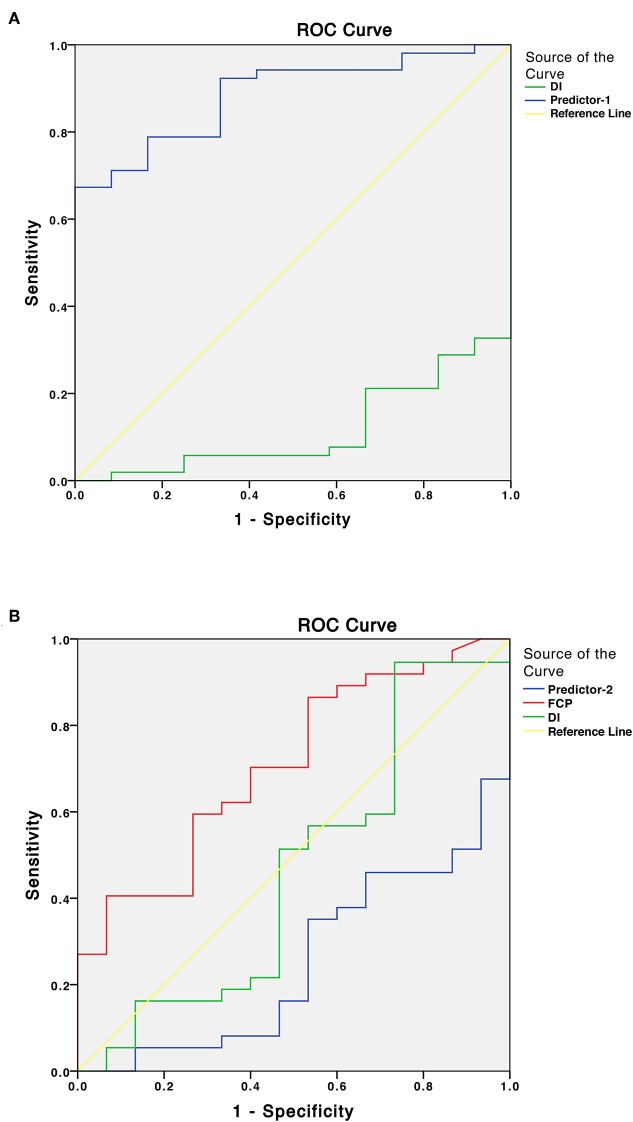
**(A)** Receiver operating characteristic (ROC) curves of the disposition index (DI) and Predictor-1 for predicting glucose intolerance (DM/IFG/IGT) before surgery. Predictor is the predicted value calculated by the prediction model using logistic regression analysis. **(B)** ROC curves of the fasting C-peptide (FCP), DI and Predictor-2 for predicting improved glucose tolerance status after surgery.

Preoperatively, 18 patients (28.1%) had DM, 34 (53.1%) had PreDM, and 12 (18.8%) had NGT (Figure 1). Supplementary Table 2 shows the three groups' preoperative clinical characteristics. Age, sex, body mass index (BMI), disease duration, random GH, nadir GH, IGF-1, and IGF-1 (%ULN) did not differ significantly, while PG, INS, CP after OGTT, and HbA1c differed significantly among the three groups. All β-cell function indices demonstrated that pancreatic β-cell functions were significantly lower in patients with DM than in those with PreDM and NGT (all *P* < 0.005). HOMA-%S, QUICKI, HOMA2-IR, and IAI did not differ significantly. However, the Matsuda index and eMCR of the DM group were significantly lower, and the HOMA1-IR of the DM group was significantly higher than that of PreDM and NGT groups (Table 1; Supplementary Table 2). IGF-1 was significantly positively correlated with HOMA1-%β (INS) and HOMA2-%β (INS) in both the DM (*r* = 0.504, *P* = 0.033 and *r* = 0.528, *P* = 0.024, respectively) and NGT groups (*r* = 0.608, *P* = 0.036 and *r* = 0.595, *P* = 0.041, respectively). IGF-I was also weakly correlated with HOMA1-%β (INS) (*r* = 0.281, *P* = 0.025) and HOMA2-%β (INS) (*r* = 0.282, *P* = 0.024) for the entire cohort. IGF-1 was significantly correlated with HOMA-IR in both the NGT and entire groups but unassociated with the HOMA-IR in the DM or PreDM group. No glucose metabolic parameters before surgery were correlated with disease duration, random GH, nadir GH, or IGF-1 (%ULN) in our study (Supplementary Table 3). To determine the risk factors associated with glucose intolerance before surgery, we performed multivariate logistic regression analysis. DI (OR = 0.609, 95%CI 0.451–0.823, *P* = 0.001) and Predictor-1 (OR = 5.120, 95%CI 1.634–16.041, *P* = 0.002) were determined to predict glucose intolerance. The prediction model formula calculated using logistic regression was Predictor-1 = 1/ (1+e^−Z^), *Z* = 3.128–0.496 × DI. The ROC was then analyzed to determine the predictive values of DI and Predictor-1 (Table 2; Figure 2A). DI was excluded due to its small AUC (0.115). The optimal cut-off value of Predictor-1 was 0.866, with 71.2% sensitivity and 91.7% specificity.

**Table 1:** Comparisons of preoperative, immediately postoperative, and 3-month postoperative parameters among DM, PreDM, and NGT group.

	**DM (*****n*** **=** **18)**				**PreDM (*****n*** **=** **34)**				**NGT (*****n*** **=** **12)**			
	**Preop**.	**Immediately postop**.	**3-month postop**.	***P* value**	**Preop**.	**Immediately postop**.	**3–month postop**.	***P* value**	**Preop**.	**Immediately postop**.	**3-month postop**.	***P* value**
Random GH (μg/L)	15.0 (10.4–45.7)	2.7 (1.6–8.2)^*^	2.3 (1.1–4.5)^*^	**0.000**	15.6 (8.9–35.6)	1.6 (0.8–3.5)^*^	2.2 (0.4–4.4)^*^	**0.000**	14.1 (8.8–49.1)	1.8 (0.8–3.7)^*^	0.6 (0.3–4.0)^*^	**0.000**
Nadir GH (μg/L)	13.0 (8.3–21.6)	2.0 (1.0–6.3)^*^	1.3 (0.2–2.5)^*^^∧^	**0.000**	10.7 (5.1–31.2)	0.9 (0.4–2.6)^*^	0.4 (0.1–1.8)^*^^∧^	**0.000**	12.9 (8.5–37.6)	0.8 (0.6–1.9)^*^	0.4 (0.1–1.6)^*^^∧^	**0.000**
IGF-1 (μg/L)	922.0 (755.8–1091.5)	702.5 (573.8–919.0)^*^	567.0 (283.3–630.8)^*^^∧^	**0.000**	859.5 (719.0–1025.5)	610.0 (484.0–814.5)^*^	302.0 (239.8–484.8)^*^^∧^	**0.000**	899.0 (686.5–1022.5)	741.5 (477.3–939.3)^*^	347.5 (257.8–648.5)^*^^∧^	**0.000**
IGF-1 (%ULN)	3.5 ± 1.1	2.8 ± 1.3^*^	1.9 ± 1.0^*^^∧^	**0.000**	3.1 ± 1.0	2.4 ± 0.9^*^	1.4 ± 0.6^*^^∧^	**0.000**	2.9 ± 0.7	2.3 ± 0.7^*^	1.5 ± 0.8^*^^∧^	**0.000**
HbA1c (%)	7.1 (6.8–9.8)	6.9 (6.5–8.3)^*^	5.9 (5.5–6.0)^*^^∧^	**0.000**	5.6 (5.5–5.7)	5.5 (5.4–5.7)^*^	5.3 (5.1–5.4)^*^^∧^	**0.000**	5.6 (5.4–5.7)	5.6 (5.4–5.6)	5.3 (5.1–5.3)^*^^∧^	**0.001**
FPG (mmol/L)	7.3 (6.6–8.3)	7.5 (6.1–8.6)	6.1 (5.5–65.8)^*^^∧^	**0.002**	5.8 (5.3–6.3)	5.7 (5.4–6.3)	5.3 (4.9–5.5)^*^^∧^	**0.000**	5.3 (5.1–5.4)	5.3 (4.9–5.8)	5.3 (5.1–5.4)	0.614
2h-PG (mmol/L)	14.3 (11.5–15.9)	15.6 (13.0–16.7)	8.1 (6.3–10.7)^*^^∧^	**0.000**	8.9 (7.5–9.8)	9.3 (7.9–10.7)	5.6 (4.8–7.0)^*^^∧^	**0.000**	5.7 (5.5–6.6)	6.1 (5.3–7.7)	5.1 (4.2–5.7)^*^^∧^	**0.040**
FINS (mU/L)	15.3 (8.7–22.3)	11.4 (7.8–16.2)	8.2 (7.1–10.6)^*^^∧^	**0.003**	17.0 (12.7–23.1)	11.1 (7.8–17.7)^*^	7.5 (5.6–10.7)^*^^∧^	**0.000**	18.2 (11.0–27.6)	10.4 (8.7–19.6)	10.8 (6.0–14.6)^*^	**0.005**
INS_120_ (mU/L)	66.5 (33.6–92.0)	39.5 (28.2–78.9)	38.4 (24.6–58.7)^*^	0.154	97.2 (65.0–163.7)	97.0 (65.2–175.1)	40.2 (21.1–68.0)^*^^∧^	**0.000**	81.4 (60.5–170.1)	52.4 (39.6–93.4)	36.4 (19.3–53.1)^*^^∧^	**0.005**
FCP (ng/ml)	1.8 (1.5–3.0)	2.0 (1.3–3.0)	1.6 (1.4–2.0)	0.358	2.5 (1.8–3.2)	2.0 (1.5–2.7)	1.3 (1.1–1.7)^*^^∧^	**0.000**	2.2 (1.9–3.4)	1.7 (1.5–2.4)^*^	1.7 (1.2–2.2)^*^	0.105
CP_120_ (ng/ml)	5.4 (3.9–9.1)	5.4 (4.3–10.8)	5.7 (4.4–7.7)	0.278	9.5 (6.9–11.8)	11.4 (9.2–14.0)^*^	6.7 (4.0–7.9)^*^^∧^	**0.000**	8.5 (6.6–10.9)	9.1 (6.3–11.6)	5.8 (4.4–7.8)^*^^∧^	**0.017**
**Indices of** **β-cell function**												
HOMA1-%β (INS)	90.0 (51.2–149.3)	52.2 (40.1–88.6)	61.2 (47.4–108.3)^*^	0.179	178.8 (102.3–209.3)	100.2 (64.5–155.5)^*^	96.0 (69.0–136.6)^*^	**0.000**	202.6 (131.3–340.5)	133.6 (102.4–226.5)^*^	128.4 (66.3–233.0)^*^	**0.006**
HOMA2-%β (INS)	79.5 (54.9–119.5)	55.9 (43.8–82.7)	66.9 (54.3–98.7)	0.320	135.8 (94.5–154.1)	92.6 (69.1–124.6)^*^	90.8 (71.8–113.3)^*^	**0.000**	152.6 (113.0–215.7)	112.6 (94.6–163.2)^*^	111.0 (71.3–160.9)^*^	**0.009**
HOMA2-%β (CP)	56.9 (43.2–103.8)	65.4 (40.1–96.8)	88.5 (56.0–102.0)	0.128	111.0 (86.7–138.7)	98.9 (73.0–110.5)^*^	90.5 (73.2–104.0)^*^	**0.000**	125.6 (110.0–159.2)	114.3 (86.5–136.7)	110.9 (82.9–128.2)^*^	0.174
AUC_PG_	2191.5 (1926.0–2529.4)	2373.0 (1903.9–2547.4)	1588.5 (1451.6–1759.5)^*^^∧^	**0.000**	1544.3 (1466.6–1683.8)	1569.8 (1417.1–1848.8)	1212.8 (1035.8–1306.9)^*^^∧^	**0.000**	1190.3 (1122.4–1240.1)	1281.0 (1209.0–1369.1)	1056.0 (1002.8–1142.6)^*^^∧^	**0.001**
AUC_INS_	8588.3 (5405.5–15098.7)	5417.0 (4483.7–13508.6)	5743.7 (4614.9–7398.6)^*^	0.209	18758.5 (10128.2–27400.3)	13668.8 (9059.3–28890.2)	8285.9 (5312.1–12034.1)^*^^∧^	**0.000**	17290.6 (13478.6–32335.1)	16757.6 (12964.6–23786.0)	11551.9 (7541.5–17742.8)^*^^∧^	**0.013**
AUC_CP_	796.4 (522.6–1304.4)	777.7 (656.4–1595.2)	866.6 (689.8–1088.1)	0.358	1552.6 (1074.7–1759.7)	1474.7 (1198.0–2086.1)	974.9 (691.7–1220.1)^*^^∧^	**0.000**	1325.3 (1214.2–1796.6)	1441.1 (1278.7–1748.4)	1176.7 (928.0–1313.8)^*^^∧^	**0.002**
AUC_INS_/AUC_PG_	4.0 (1.9–7.6)	2.5 (1.7–7.1)	3.9 (2.5–6.2)	0.846	12.7 (6.3–17.0)	9.0 (5.9–14.2)^*^	7.1 (4.3–9.6)^*^^∧^	**0.000**	14.7 (11.1–25.3)	12.7 (11.3–20.1)^*^	10.4 (7.0–17.6)^*^	**0.017**
AUC_CP_/AUC_PG_	0.4 (0.2–0.7)	0.3 (0.2–0.7)	0.6 (0.4–0.8)^*^	0.179	1.0 (0.8–1.2)	1.0 (0.8–1.2)	0.8 (0.7–1.0)^*^^∧^	**0.042**	1.2 (1.0–1.5)	1.1 (1.0–1.4)	1.1 (0.8–1.3)^*^	0.472
IGI	0.3 (0.1–0.4)	0.2 (0.1–0.4)	0.3 (0.1–0.6)	0.801	1.4 (0.6–2.0)	0.9 (0.5–1.8)	0.8 (0.5–1.6)^*^	0.157	2.9 (1.8–3.8)	2.7 (1.3–3.7)	2.2 (1.1–3.5)	0.338
IGI/IR	0.04 (0.02–0.07)	0.08 (0.01–0.1)	0.1 (0.05–0.2)^*^^∧^	**0.001**	0.3 (0.2–0.5)	0.3 (0.2–0.5)	0.4 (0.3–0.7)^*^^∧^	**0.002**	0.5 (0.4–0.8)	0.5 (0.4–1.5)	0.8 (0.4–1.3)	0.558
Disposition Index	0.5 (0.3–0.8)	0.8 (0.1–1.3)	1.2 (0.7–1.9)^*^^∧^	**0.005**	2.4 (1.5–3.4)	2.6 (1.6–3.6)	4.1 (2.4–6.4)^*^^∧^	**0.000**	5.5 (3.1–6.8)	4.6 (3.4–8.1)	6.8 (3.9–9.6)^*^	0.264
ISSI2	8.2 (5.5–10.2)	8.8 (4.6–13.1)	17.8 (13.3–23.3)^*^^∧^	**0.000**	23.3 (18.2–28.6)	24.8 (17.7–31.2)	33.7 (25.8–40.1)^*^^∧^	**0.000**	29.7 (27.9–38.5)	30.2 (23.9–49.5)	41.0 (33.0–46.8)^*^	**0.017**
MBCI	7.4 (3.3–8.2)	4.1 (2.8–7.5)	4.0 (2.7–6.7)^*^	0.115	8.1 (5.5–12.2)	4.9 (3.5–7.4)^*^	6.3 (3.7–9.8)	**0.028**	14.3 (9.9–19.9)	6.7 (5.4–11.7)	10.2 (5.7–17.8)	0.174
eFPIS (pmol/L)	806.4 (214.2–1187.0)	422.1 (71.5–825.2)	694.9 (112.7–912.1)	0.311	1757.4 (1168.3–2389.8)	1270.5 (847.8–2156.9)^*^	1086.9 (786.3–1431.6)^*^^∧^	**0.000**	3185.5 (1477.4–4321.4)	2141.1 (1532.4–2900.3)^*^	1980.6 (998.5–3467.8)^*^^∧^	**0.046**
eSPIS (pmol/L)	234.1 (105.9–326.2)	154.3 (82.4–240.2)	202.1 (89.9–263.9)	0.249	441.8 (327.8–605.8)	344.8 (242.2–544.8)^*^	288.2 (216.1–371.9)^*^^∧^	**0.000**	775.0 (377.8–1036.6)	524.9 (394.4–710.1)	491.6 (266.4–836.3)^*^	**0.039**
**Indices of insulin sensitivity**												
HOMA1-%S (INS)	16.3 (11.9–27.7)	29.6 (14.5–45.8)^*^	44.1 (32.0–58.6)^*^^∧^	**0.002**	22.2 (16.3–31.7)	36.6 (23.4–51.9)^*^	59.9 (39.1–79.2)^*^^∧^	**0.000**	23.3 (16.9–39.1)	38.3 (24.1–51.7)^*^	43.4 (31.3–69.9)^*^	**0.002**
HOMA2-%S (INS)	37.4 (28.1–71.0)	64.4 (39.7–91.5)^*^	88.8 (69.3–101.8)^*^^∧^	**0.002**	43.7(32.6–59.0)	67.7 (43.6–97.1)^*^	101.4 (70.8–135.5)^*^^∧^	**0.000**	42.5 (30.1–69.8)	71.8 (42.7–89.2)^*^	72.7 (53.7–125.0)^*^	**0.002**
HOMA2-%S (CP)	56.6 (40.7–75.2)	58.9 (42.2–89.2)	74.3 (63.7–90.5)^*^^∧^	**0.042**	52.1(43.1–70.2)	66.5 (48.6–86.8)	104.8 (82.2–119.5)^*^^∧^	**0.000**	62.2 (39.4–71.2)	79.4 (56.1–92.6)	81.1 (62.9–116.2)^*^	0.105
QUICKI	0.47 (0.44–0.52)	0.53 (0.46–0.59)^*^	0.59 (0.50–0.63)^*^^∧^	**0.002**	0.50 (0.47–0.54)	0.56 (0.50–0.61)^*^	0.63 (0.55–0.69)^*^^∧^	**0.000**	0.50 (0.47–0.57)	0.57 (0.50–0.61)^*^	0.58 (0.54–0.66)^*^	**0.002**
Matsuda Index (WBISI)	1.8 (1.5–3.4)	2.8 (1.8–4.9)	4.6 (3.2–5.4)^*^^∧^	**0.003**	2.0 (1.5–2.6)	2.8 (1.7–4.2)^*^	5.5 (3.4–7.6)^*^^∧^	**0.000**	2.2 (1.3–3.3)	2.6 (2.1–3.7)^*^	3.5 (2.7–6.3)^*^^∧^	**0.001**
eMCR (ml/kg/min/)	6.2 (5.3–7.1)	5.9 (5.2–6.9)	8.3 (7.1–8.9)^*^^∧^	**0.000**	8.8 (7.6–9.6)	8.3 (7.7–9.2)	9.9 (9.3–10.5)^*^^∧^	**0.000**	9.8 (8.9–10.3)	10.0 (9.0–10.5)	10.3 (9.4–11.0)^*^^∧^	0.105
**Indices of insulin resistance**												
HOMA1-IR (INS)	6.2 (3.6–8.4)	3.4 (2.2–6.9)	2.3 (1.7–3.2)^*^^∧^	**0.002**	4.5 (3.2–6.1)	2.7 (1.9–4.3)^*^	1.7 (1.3–2.6)^*^^∧^	**0.000**	4.3 (2.6–6.2)	2.6 (2.0–4.6)	2.3 (1.4–3.2)^*^	**0.002**
HOMA2-IR (INS)	2.7 (1.4–3.6)	1.6 (1.1–2.5)	1.1 (1.0–1.4)^*^^∧^	**0.002**	2.3 (1.7–3.1)	1.5 (1.0–2.3)^*^	1.0 (0.7–1.4)^*^^∧^	**0.000**	2.4 (1.4–3.5)	1.4 (1.1–2.5)	1.4 (0.8–1.9)^*^	**0.002**
HOMA2-IR (CP)	1.8 (1.3–2.5)	1.7 (1.1–2.4)	1.3 (1.1–1.6)^*^^∧^	**0.042**	1.9 (1.4–2.3)	1.5 (1.2–2.1)	1.0 (0.8–1.2)^*^^∧^	**0.000**	1.6 (1.4–2.5)	1.3 (1.1–1.8)^*^	1.2 (0.9–1.6)^*^	0.098
IAI	0.0072(0.0053–0.012)	0.013 (0.0065–0.02)^*^	0.020 (0.01–0.03)^*^^∧^	**0.002**	0.0099 (0.0073–0.014)	0.017 (0.010–0.023)^*^	0.026 (0.016–0.035)^*^^∧^	**0.000**	0.010 (0.0075–0.017)	0.017 (0.011–0.023)^*^	0.019 (0.014–0.031)^*^	**0.002**

*TC, total cholesterol; TG, total triglycerides; GH, growth hormone; IGF-1, insulin-like growth factor-1; ULN, upper limit of normal; HbA1c, glycosylated hemoglobin; FPG, fasting plasma glucose; FINS, fasting insulin; FCP, fasting C-peptide; HOMA-%β, homeostasis assessment model of β-cell function; AUC, areas under the curve; IGI, insulinogenic index; DI, disposition index; ISSI2, the OGTT insulin secretion sensitivity index 2; MBCI, modified β-cell function index; eFPIS, estimated first phase insulin release; eSPIS, estimated second phase insulin release; HOMA-%S, homeostasis assessment model of insulin sensitivity; QUICKI, quantitative insulin sensitivity check index; WBISI, whole body insulin sensitivity index; eMCR, estimated metabolic clearance rate of glucose; HOMA-IR, homeostasis assessment model of insulin resistance; IAI, insulin activity index*.

*P values are for variations among the preoperative, immediately postoperative and 3-month postoperative groups*.

* *Means that p < 0.05 vs. the preoperative group*.

∧ *Means that p < 0.05 for immediately postoperative group vs. 3-month postoperative group*.

*Bold values means P < 0.05*.

**Table 2:** ROC analysis of baseline parameters for predicting glucose intolerance (DM/IFG/IGT) before surgery, and parameters for predicting improvement of glucose tolerance status after surgery.

**Parameters**	**AUC**	***P* value**	**95%CI**	**Cut–off value**	**Sensitivity**	**Specificity**	**PPV**	**NPV**
**ROC analysis of risk factors for predicting glucose intolerance (dm/ifg/igt) before surgery**								
Disposition Index (DI)	0.115^*^	0.000	0.030–0.201	–	–	–	–	–
Predictor−1	0.885	0.000	0.799–0.970	0.866	71.2%	91.7%	97.4%	42.3%
**ROC analysis of parameters for predicting improvement of glucose tolerance status after surgery**								
FCP (ng/ml)	0.709	0.019	0.558–0.860	2.445	69.5%	89.2%	86.6%	74.5%
Disposition Index (DI)	0.465^*^	0.046	0.273–0.656	–	–	–	–	–
Predictor−2	0.252^*^	0.005	0.112–0.393	–	–	–	–	–

*AUC, areas under the curve; CI, confidence interval; PPV, positive predictive value; NPV, negative predictive value; FCP, fasting C-peptide*.

* *These parameters were excluded due to their AUC < 0.5*.

*Predictor is the predicted value calculated by the prediction model using logistic regression analysis*.

### Postoperative Glucose Tolerance Status and Glucose Metabolic Parameters

Five patients (7.8%) had DM, 19 (29.7%) had PreDM, and 40 (62.5%) had NGT 3 months after surgery (Figure 1). Table 1 compares the preoperative, immediate postoperative and 3-month postoperative parameters among the three groups. Random and nadir GH, IGF-1, IGF-1 (%ULN), HbA1c, 2h-PG and FINS decreased significantly after surgery in all groups. The insulin sensitivity indices were all significantly elevated, and IR was significantly reduced 3 months after surgery regardless of preoperative glucose tolerance status (Table 1). For the entire cohort, ΔIGF-1 and ΔIGF-1 (%ULN), which indicate the parameter changes before and after surgery, respectively, were weakly correlated with ΔHOMA2-%S (INS) (*r* = −0.256, *P* = 0.041 and *r* = −0.274, *P* = 0.029, respectively), ΔHOMA2-%S (CP) (*r* = −0.236, *P* = 0.048 and *r* = −0.257, *P* = 0.040, respectively), and the ΔMatsuda index (*r* = 0.339, *P* = 0.006). ΔRandom and nadir GHs did not correlate with the Δparameters of glucose metabolism (Supplementary Table 4).

### Parameters Associated With Improved Glucose Tolerance After Surgery

**Figure 3:**
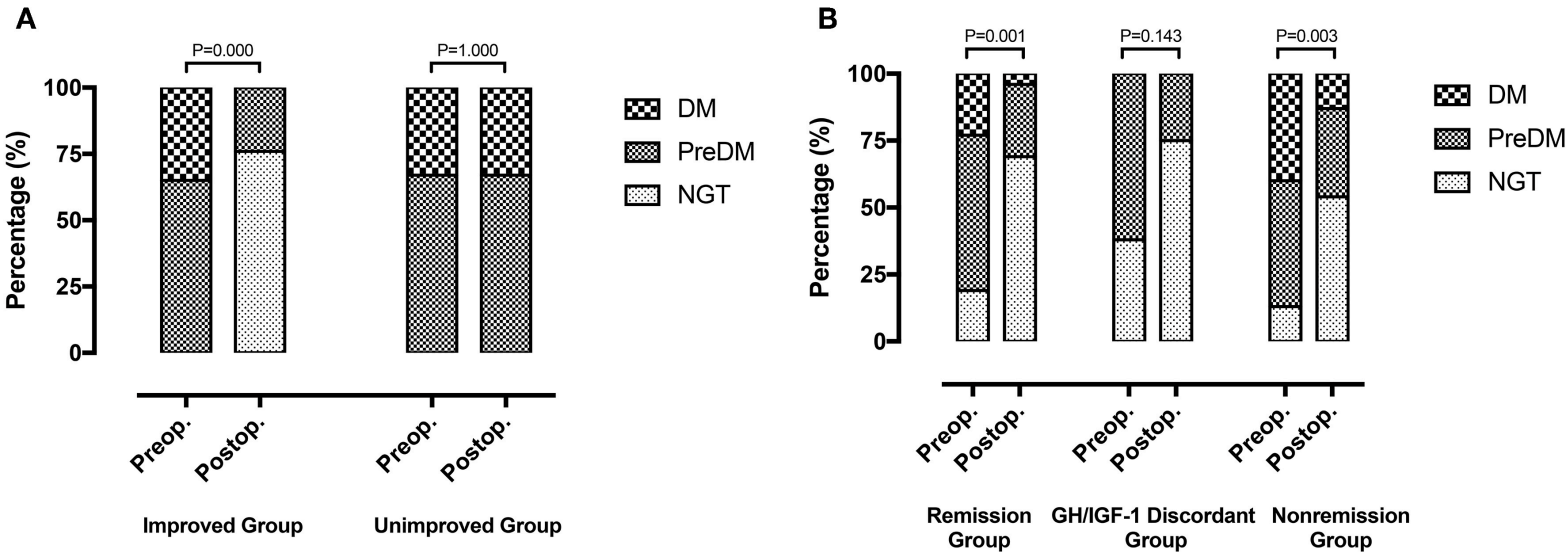
**(A)** Improved group (*n* = 37): 13 DM patients and 24 PreDM patients before surgery, 9 PreDM patients and 28 NGT patients after surgery (*P* = 0.000). Unimproved group (*n* = 15): 5 DM patients and 10 PreDM patients before and after surgery (*P* = 1.000). **(B)** Remission group (*n* = 26): 6 DM patients, 15 PreDM patients and 5 NGT patients before surgery; 1 DM patient, 7 PreDM patients, and 18 NGT patients after surgery (*P* = 0.001). GH/IGF-1 discordant group (*n* = 8): 5 PreDM patients and 3 NGT patients before surgery; 2 PreDM patients and 6 NGT patients after surgery (*P* = 0.143). Non-remission group (*n* = 30): 12 DM patients, 14 PreDM patients, and 4 NGT patients before surgery; 4 DM patients, 10 PreDM patients and 16 NGT patients after surgery (*P* = 0.003).

Fifty-two patients (81.3%) with preoperative abnormal glucose tolerance statuses were classified as the improved (13 with DM and 24 with PreDM) and unimproved groups (5 with DM and 10 with PreDM). Postoperatively, 9 patients had PreDM and 28 had NGT in the improved group, while 5 had DM and 10 had PreDM in the unimproved group (Figure 3A).

Table 3 compares the baseline parameters between the two groups. Age, sex, BMI, random GH, nadir GH, IGF-1, IGF-1 (%ULN), ratio of preoperative glucose tolerance, HbA1c, PG, INS_120_, and CP_120_ did not differ significantly between the improved and unimproved groups. However, patients in the unimproved group had longer disease durations (*P* = 0.046), lower FINS (*P* = 0.028), and lower FCP (*P* = 0.019) than did those in the improved group. Regarding the β-cell function indices, HOMA-%β (INS) and DI were significantly higher in the improved group, but the other indices did not differ significantly between the two groups. Patients in the improved group showed significantly higher IR and lower insulin sensitivity than did the unimproved group. Postoperatively, patients in the improved group had lower PG and AUC_PG_ and higher ISSI2 and eMCR than did those in the unimproved group.

**Table 3:** Comparisons of baseline and 3-month postoperative parameters of patients with preoperative abnormal glucose tolerance status (*n* = 52) between the improved and unimproved groups.

**Parameters**	**Preoperation**			**3–month Postoperation**		
	**Improved (*n* = 37)**	**Unimproved (*n* = 15)**	***P* value**	**Improved (*n* = 37)**	**Unimproved (*n* = 15)**	***P* value**
Age (years)	41.2 ± 9.4	42.5 ± 16.0	0.887	–	–	–
Sex (male:female)	19:18	9:6	0.571	–	–	–
Body mass index (kg/m^2^)	26.3 ± 4.0	25.8 ± 3.5	0.694	–	–	–
Disease duration (yrs)	5.7 ± 3.8	8.5 ± 5.5	**0.046**	–	–	–
Random GH (μg/L)	22.0 (8.9–50.6)	11.1 (7.6–19.0)	0.130	1.6 (0.4–5.1)^*^	2.6 (1.1–4.3)^∧^	0.578
Nadir GH (μg/L)	13.7 (6.1–30.3)	9.0 (5.3–11.9)	0.182	0.6 (0.2–1.9)^*^	0.4 (0.1–2.0)^∧^	0.840
IGF−1 (μg/L)	890.0 (758.0–1040.0)	926.0 (633.5–1093.5)	0.724	323.0 (237.0–564.0)^*^	443.0 (291.5–628.0)^∧^	0.280
IGF−1 (%ULN)	3.3 ± 1.1	3.1 ± 1.0	0.485	1.5 ± 0.8^*^	1.8 ± 0.9^∧^	0.369
Remission Status of acromegaly	–	–	–	16:3:18 (remission: GH/IGF−1 discordance: non-remission)	5:2:8 (remission: GH/IGF−1 discordance: non-remission)	0.629
Glucose tolerance status (DM: PreDM: NGT)	13:24:0	5:10:0	0.902	0:9:28^*^	5:10:0	**0.000**
HbA1c (%)	5.7 (5.6–6.6)	6.0 (5.6–7.0)	0.707	5.3 (5.2–5.5)^*^	5.5 (5.2–6.1)^∧^	0.490
FPG (mmol/L)	6.1 (5.6–6.8)	6.3 (5.5–6.9)	0.808	5.3 (4.9–5.5)^*^	5.8 (5.3–6.4)	**0.023**
2h–PG (mmol/L)	9.9 (8.2–11.4)	9.6 (8.7–13.1)	0.777	5.5 (4.9–6.8)^*^	9.6 (8.1–11.1)	**0.000**
FINS (mU/L)	21.3 (12.7–26.5)	12.8 (8.5–18.0)	**0.028**	7.4 (6.0–10.7)^*^	8.2 (6.3–11.6)^∧^	0.473
INS_120_ (mU/L)	89.0 (65.0–118.7)	60.2 (35.8–120.1)	0.113	42.2 (19.8–63.8)^*^	38.6 (28.0–83.4)^∧^	0.358
FCP (ng/ml)	2.5 (1.8–3.3)	1.8 (1.5–2.5)	**0.019**	1.4 (1.2–1.8)^*^	1.4 (1.2–2.0)	0.936
CP_120_ (ng/ml)	9.1 (6.7–10.6)	5.7 (4.0–10.7)	0.196	6.1 (4.4–7.4)^*^	7.0 (4.0–8.5)	0.579
**Indices of** **β-cell function**						
HOMA1-%β (INS)	158.1 (94.6–206.3)	91.6 (50.5–179.2)	**0.046**	89.6 (63.2–110.2)^*^	65.2 (49.2–131.8)	0.254
HOMA2-%β (INS)	124.5 (85.5–153.5)	76.0 (55.1–140.3)	**0.041**	86.5 (68.8–100.5)^*^	69.6 (54.9–110.0)	0.226
HOMA2-%β (CP)	108.1 (71.4–127.6)	69.0 (50.9–127.2)	0.054	92.7 (77.6–104.4)^*^	72.4 (56.9–102.3)	0.132
AUC_PG_	1677.0 (1509.0–2002.5)	1695.5 (1503.0–2264.3)	0.607	1224.0 (1044.0–1450.5)^*^	1645.5 (1287.8–1881.8)	**0.002**
AUC_INS_	15383.1 (9279.8–20247.8)	10385.7 (5206.3–21955.4)	0.461	6345.3 (4880.9–11189.4)^*^	7922.9 (4553.1–11854.1)^∧^	0.754
AUC_CP_	1310.0 (956.7–1631.3)	755.4 (530.3–1629.9)	0.254	881.3 (692.7–1161.5)^*^	941.4 (665.9–1184.1)	0.960
AUC_INS_/AUC_PG_	8.7 (5.5–14.1)	6.0 (2.2–16.0)	0.380	5.2 (3.5–8.5)^*^	6.5 (2.3–9.6)	0.635
AUC_CP_/AUC_PG_	0.8 (0.5–1.1)	0.4 (0.2–1.1)	0.348	0.7 (0.6–0.9)	0.7 (0.4–1.0)^∧^	0.391
IGI	0.7 (0.4–1.6)	0.6 (0.2–1.7)	0.816	0.6 (0.4–0.9)	0.7 (0.2–1.3)	0.579
IGI/IR	0.2 (0.05–0.3)	0.2 (0.06–0.5)	0.621	0.3 (0.2–0.6)	0.2 (0.1–0.5)	0.193
Disposition Index	1.6 (0.7–3.3)	1.2 (0.8–2.6)	**0.048**	3.3 (2.0–5.3)^*^	1.6 (1.1–4.0)	0.108
ISSI2	18.5 (10.3–25.4)	18.1 (8.4–28.0)	0.896	28.1 (23.4–35.3)^*^	19.7 (12.9–33.0)^∧^	**0.031**
MBCI	7.9 (5.5–11.5)	5.5 (3.4–8.6)	0.132	5.9 (3.5–9.0)^*^	4.1 (2.2–8.3)	0.237
eFPIS (pmol/L)	1370.2 (853.6–1987.8)	900.9 (378.5–1909.8)	0.369	907.9 (658.4–1199.3)^*^	1045.8 (155.1–1420.8)	0.960
eSPIS (pmol/L)	365.2 (243.9–526.9)	280.0 (134.5–482.0)	0.348	246.3 (190.1–311.6)^*^	279.7 (96.2–368.9)	0.912
**Indices of insulin sensitivity**						
HOMA1-%S (INS)	18.0 (12.8–26.7)	26.3 (22.4–36.4)	**0.028**	53.1 (38.1–75.9)^*^	41.6 (27.7–71.1)^∧^	0.280
HOMA2-%S (INS)	36.4 (28.6–57.9)	56.7 (42.4–77.9)	**0.038**	101.8 (72.4–131.4)^*^	95.1 (61.6–123.2)^∧^	0.348
HOMA2-%S (CP)	49.4 (37.9–69.6)	61.6 (53.3–78.3)	**0.031**	95.6 (73.1–115.6)^*^	88.9 (62.1–117.5)	0.679
QUICKI	0.48 (0.45–0.52)	0.52 (0.50–0.56)	**0.028**	0.61 (0.56–0.66)^*^	0.58 (0.52–0.67)^∧^	0.391
Matsuda Index (WBISI)	1.7 (1.4–2.6)	2.1 (1.8–3.9)	0.108	5.4 (3.6–7.7)^*^	4.2 (3.0–5.4)^∧^	0.096
eMCR (ml.kg^−1^.min^−1^)	8.1 (7.0–9.4)	7.6 (6.5–9.3)	0.896	9.8 (8.9–10.6)^*^	9.2 (7.2–9.4)	**0.004**
**Indices of insulin resistance**						
HOMA1-IR (INS)	5.5 (3.7–7.8)	3.8 (2.7–4.5)	**0.028**	1.9 (1.3–2.6)^*^	2.4 (1.4–3.6)^∧^	0.280
HOMA2-IR (INS)	2.8 (1.7–3.5)	1.8 (1.3–2.4)	**0.037**	1.0 (0.8–1.4)^*^	1.1 (0.8–1.6)^∧^	0.342
HOMA2-IR (CP)	2.0 (1.4–2.64)	1.6 (1.3–1.9)	**0.031**	1.1 (0.9–1.4)^*^	1.1 (0.9–1.6)	0.686
IAI	0.008 (0.006–0.012)	0.012 (0.001–0.017)	**0.028**	0.023 (0.017–0.031)^*^	0.018 (0.012–0.032)^∧^	0.308

*TC, total cholesterol; TG, total triglycerides; GH, growth hormone; IGF-1, insulin-like growth factor-1; ULN, upper limit of normal; HbA1c, glycosylated hemoglobin; FPG, fasting plasma glucose; FINS, fasting insulin; FCP, fasting C-peptide; HOMA-%β, homeostasis assessment model of β-cell function; AUC, areas under the curve; IGI, insulinogenic index; DI, disposition index; ISSI2, the OGTT insulin secretion sensitivity index 2; MBCI, modified β-cell function index; eFPIS, estimated first phase insulin release; eSPIS, estimated second phase insulin release; HOMA-%S, homeostasis assessment model of insulin sensitivity; QUICKI, quantitative insulin sensitivity check index; WBISI, whole body insulin sensitivity index; eMCR, estimated metabolic clearance rate of glucose; HOMA-IR, homeostasis assessment model of insulin resistance; IAI, insulin activity index*.

*P values are for variations between the improved and unimproved group*.

* *Means that p < 0.05 between the preoperative and 3-month postoperative parameters in the improved group*.

∧ *Means that p < 0.05 between the preoperative and 3-month postoperative parameters in the unimproved group*.

*Bold values means P < 0.05*.

**Figure 4:**
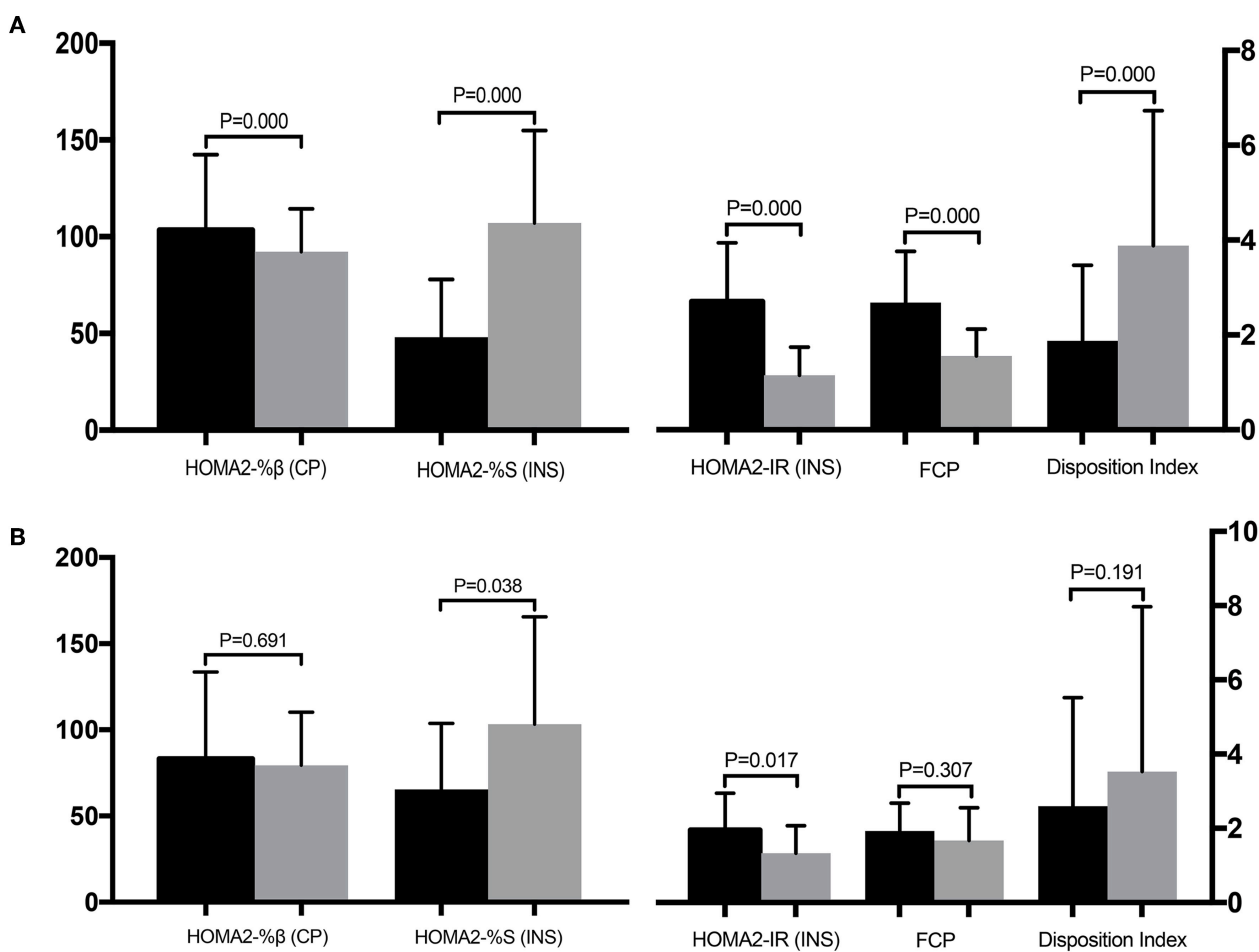
Comparisons of the clinical parameters before (black column) and after (gray column) surgery for the improved **(A)** and unimproved **(B)** groups. Pancreatic β-cell function is represented by HOMA2-%β (CP), insulin sensitivity is represented by HOMA2-%S (INS), and insulin resistance is represented by HOMA2-IR (INS).

Table 3 compares the clinical parameters before and after surgery for the improved and unimproved groups. All parameters except the AUC_CP_/AUC_PG_, IGI, and IGI/IR, differed significantly before and after surgery in the improved group (Figure 4A). However, in the unimproved group, PG, CP, HOMA-%β, DI, HOMA2-%S (CP), and HOMA2-IR (CP) did not differ significantly compared with the preoperative values. INS, AUC_INS_, and HOMA-IR (INS) were significantly decreased after surgery in the unimproved group, while HOMA-%S (INS), QUICKI, the Matsuda index, and IAI were significantly elevated after surgery (Figure 4B).

To determine the factors associated with improved glucose intolerance after surgery, we performed multivariate logistic regression analysis including all baseline parameters and remission statuses of the patients with acromegaly. FCP (OR = 2.639, 95%CI 1.149–6.024, *P* = 0.022), DI (OR = 1.397, 95%CI 0.969–2.014, *P* = 0.043) and Predictor-2 (OR = 0.578, 95%CI 0.359–0.891, *P* = 0.035) were determined to predict improved glucose tolerance after surgery. The prediction model formula calculated using logistic regression was Predictor-2 = 1/ (1+e-Z), *Z* = 1.291–0.969 × FCP+0.335 × DI. The ROC was analyzed to determine the predictive values of FCP, DI and Predictor-2 (Table 2; Figure 2B). DI and Predictor-2 were excluded due to their small AUCs (0.465 and 0.252, respectively). The optimal cut-off FCP was 2.445 ng/ml, with 69.5% sensitivity, and 89.2% specificity.

### Glucose Metabolism Among the Remission, GH/IGF-1 Discordant, and Non-remission Groups After Surgery

At the last follow-up, patients were divided into 3 groups based on their acromegaly remission statuses after surgery: the remission group (26/64, 40.6%), the GH/IGF-1 discordant group (8/64, 12.5%), and the non-remission group (30/64, 46.9%). Preoperatively, 6 patients (23.1%) had DM, 15 (57.7%) had PreDM, and 5 (19.2%) had NGT in the remission group; 5 (62.5%) had PreDM and 3 (37.5%) had NGT in the GH/IGF-1 discordant group; and 12 (40.0%) had DM, 14 (46.7%) had PreDM, and 4 (13.3%) had NGT in the non-remission group (Figure 3B). The preoperative glucose tolerance status ratio (DM: PreDM: NGT) did not differ significantly between the remission, GH/IGF-1 discordant and non-remission groups (*P* = 0.330). After surgery, the proportions of DM, PreDM, and NGT were 3.8% (1), 26.9% (7), and 69.2% (18) in the remission group and 13.3% (4), 33.3% (10), and 53.3% (16) in the non-remission group. Glucose tolerance status was improved in both the remission (*P* = 0.001) and non-remission (*P* = 0.003) groups regardless of acromegaly remission status (Figure 3B). Two patients (25.0%) had PreDM, and 6 (75.0%) had NGT in the GH/IGF-1 discordant group after surgery, which was similar to the proportions before surgery (*P* = 0.143).

Supplementary Table 5 compares the preoperative and postoperative glucose metabolic parameters among the remission, GH/IGF-1 discordant and non-remission groups. Preoperatively, patients in the remission group had higher FINS (*P* = 0.030) and ISSI2 (*P* = 0.015) than did the other two groups. The remission group had higher HOMA-%S (*P* < 0.05), QUICKI (*P* = 0.014), Matsuda index (*P* = 0.024), eMCR (*P* = 0.024), and IAI (*P* = 0.014) values and a lower HOMA-IR (*P* < 0.05) than did the non-remission group. After surgery, random GH, nadir GH, IGF-1, IGF-1 (%ULN), HbA1c, PG, INS, and CP were decreased significantly in all groups. For the β-cell function indices, AUC_PG_, AUC_CP_, eFPIS, and eSPIS were significantly decreased, while DI and ISSI2 were significantly elevated postoperatively among the 3 groups. All indices of insulin sensitivity, including HOMA-%S, QUICKI, the Matsuda index and eMCR, were significantly increased, while HOMA-IR was significantly decreased among all groups after surgery.

## Discussion

Abnormal glucose metabolism is thought to be one of the most common complications of acromegaly (1). The chronic excess of both GH and IGF-1 plays an integral role in the intermediate metabolism impairing glucose homeostasis (4–6). In this study, based on the 2019 ADA practice guidelines for diagnosing diabetes, 28.1% of acromegaly patients had diabetes, and 53.1% had prediabetes, which is similar to data from previous studies. The literature reports the DM and PreDM prevalences as being 12–56 and 16–54%, respectively, in patients with acromegaly (2–7). Several studies reported an association between diabetes and increased cardiovascular risk and mortality among acromegaly patients (26, 27). Hence, the risk factors, predictors and therapeutic strategies for abnormal glucose metabolism in patients with acromegaly must be studied.

The pathophysiology of abnormal glucose tolerance caused by active acromegaly is complicated and inconclusive. The most important mechanism is currently believed to be insulin resistance related to GH/IGF-1 excess (4–7). GH promotes hepatic and peripheral IR, while IGF-1 reduces IR and improves insulin sensitivity. Consequently, high IGF-1 levels still fail to counteract the GH's damage to the glucose metabolism. Then, pancreatic β-cell function will be impaired due to IR-related β-cell exhaustion. In this study, GH and IGF-1 did not differ significantly among DM, PreDM and NGT patients, but INS, CP, pancreatic islet β-cell functions and insulin sensitivity were significantly lower in diabetic patients than in those with NGT. Our findings are consistent with previous studies that demonstrated impaired β-cell function, high insulin resistance, and decreased insulin sensitivity only in patients with abnormal glucose tolerance, but β-cell function was preserved in patients with NGT (8, 28). Unfortunately, because our study was a cross-sectional retrospective research, the preoperative glucose metabolic parameters of acromegalic patients demonstrated the abnormalities in β-cell function, IR and insulin sensitivity have occurred at the time of admission into hospital. We cannot judge the order in which they appeared. We can only find that the β-cell function and insulin sensitivity of DM patients were significantly worse, and IR was significantly higher than those of PreDM and NGT patients. In addition, there was no significant difference in β-cell function, IR and insulin sensitivity between the PreDM and NGT patients. Therefore, we cannot directly conclude from the current data that IR or abnormalities of insulin secretion plays a major role in GH-induced DM. But we believe that IR, insulin sensitivity and β-cell function are complementary in the development of acromegalic glucose intolerance, and collectively contribute to the glucose metabolism alterations. Similar to several previous studies, our study supported a stronger correlation between IGF-1 and insulin sensitivity and IR, but not GH, for the entire cohort, possibly because IGF-1 is a better marker of the 24-h GH secretion and metabolic profile than is GH (29, 30). In addition, IGF-1 was significantly correlated with IR in patients with NGT but not in patients with abnormal glucose tolerance, possibly due to HOMA's limited value for predicting IR calculated by a wide FPG range in patients with glucose intolerance (31, 32). Fukuoka et al. (33) detected a weak correlation between IGI and IGF-1 levels. We also found a significant positive correlation between β-cell function and IGF-1 in patients with glucose intolerance as well as in those with NGT (33). This finding suggests that IGF-1 may be a protective factor for β-cell function via lowering IR, preventing IR-related β-cell exhaustion, and improving β-cell functions (34). However, similar to the studies of Kasayama et al. (28) and Kinoshita et al. (8), we also found no correlation between GH and β-cell function, possibly because they are not linearly correlated (33).

Transsphenoidal surgery is the first-line treatment for GH-secreting pituitary adenomas (1). Successful surgical removal of somatotroph adenomas is believed to improve impaired glucose metabolism due to acromegaly (35). As reported in the literature, glucose metabolism can be restored in 23–58% of acromegaly patients with preoperative diabetes after surgically curing acromegaly (2–8). In this study, 53.8% of patients [28/52] with preoperative glucose intolerance had their glucose tolerance restored after surgery, which is consistent with previous studies. For glucose metabolism, regardless of normal or abnormal preoperative glucose tolerance status, insulin sensitivity was significantly improved and IR was significantly decreased after surgery, while the changes in β-cell function indices varied between patients with glucose intolerance and those with NGT before and after surgery. Previous studies on pre- and postoperative changes in β-cell function yielded controversial results. Kinoshita et al. (8) reported that β-cell functioning was decreased in patients with NGT before and after surgery but did not change in patients with glucose intolerance after successful surgery. However, Ronchi et al. (36) and Tzanela et al. (37) reported that the change in HOMA-β was not significant in patients with NGT or in those with glucose intolerance who were surgically cured. In this study, we found that HOMA-β was significantly decreased after surgery in patients with NGT, but postoperative HOMA-β was decreased in DM patients (statistically insignificant). This may have been due to IR-related β-cell exhaustion being terminated when the IR decreased after surgery, so the postoperative β-cell function declined correspondingly (34). For the entire cohort, changes in IGF-1 and IGF-1 (%ULN) before and after surgery were negatively correlated with the insulin sensitivity indices, indicating that the more IGF-1 decreased after surgery, the greater the improvement in insulin sensitivity. However, no correlation was found between the changes in GH before and after surgery or in any indices of glucose metabolic parameters. Afterwards, in terms of disease control in acromegaly, IR and β-cell functions decreased and insulin sensitivity increased after surgery regardless of whether acromegaly remission was achieved, which has also been reported in other studies (8, 38). This is due to the notable reductions in GH and IGF-1 after tumor debulking, whether the tumor is totally or partially resected, and will cause decreased IR and elevated insulin sensitivity, thus gradually easing the IR-induced β-cell hyperfunction (7, 39).

Subsequently, we explored the factors associated with the improved glucose tolerance after surgery in acromegaly patients. Patients whose glucose intolerance improved after surgery had shorter disease durations, lower insulin sensitivities, higher IR, and higher FINS, FCP, HOMA-β, and disposition indices, indicating that β-cell functioning was partially preserved in the improved group. These parameters may help predict the postoperative glucose tolerance improvement before treatment. Afterwards, using logistic regression and ROC analyses, the preoperative FCP (OR = 2.639) was determined to be the best independent predictor of improved glucose tolerance status after surgery in acromegaly patients. A preoperative FCP of 2.445 ng/ml is the optimal cut-off value for this prediction. CP is an enzymatic cleavage product that forms when proinsulin is transformed to insulin. CP and insulin are secreted from islet β-cells at a 1:1 concentration. CP is considered an excellent marker of endogenous insulin because it is unaffected by exogenous insulin or insulin antibodies. CP also has a higher plasma concentration than insulin and is less affected by other substances such as proinsulin. CP is commonly used to evaluate pancreatic β-cell functions in diabetic patients, while CP is slightly less accurate than insulin when evaluating IR (40–42). Based on the reliability value of CP in evaluating glucose metabolism and the high sensitivity (69.5%) and specificity (89.2%) of FCP in predicting improved glucose intolerance in this study, we believe that preoperative FCP reliably predicts surgical benefits in acromegaly patients with impaired glucose metabolism, with an 86.6% positive predictive value (PPV) and a 74.5% negative predictive value (NPV). Previous studies found some possible predictors, but no consensus was reached, possibly due to different inclusion criteria among studies and differences in diagnostic criteria for glucose intolerance, surgical outcomes, follow-up times, and other factors, resulting in a large bias in determining predictors (8, 41, 43).

Regarding the therapeutic strategy for impaired glucose metabolism in acromegaly, no expert consensus, or guideline is currently available (3–7). Based on our institution's multidisciplinary collaboration platform, including neurosurgery, endocrinology, and neuroradiology, we developed a management strategy for abnormal glucose tolerance in acromegaly patients. For acromegaly patients with glucose intolerance that is mostly diagnosed upon admission to the hospital, oral hypoglycaemic agents or insulin should be used before surgery and should be adjusted as needed while closely monitoring the blood glucose (44, 45). For PreDM patients, the primary drugs for perioperative management are antihyperglycaemic agents. If these agents are insufficient for glycaemic control, then insulin sensitisers (thiazolidinediones) and glucagon-like peptide 1 (GLP1) receptor agonists should be considered (45). For DM patients, physicians should use the same perioperative management as is used for type 2 diabetes mellitus per the 2019 ADA guidelines (45). Postoperatively, patients should continue medication therapy guided by self-monitoring of their blood glucose (SMBG). At 3 months after surgery, after reassessing the glucose metabolism based on OGTT and HbA1c, management should be adjusted for patients with altered glucose tolerance statuses. Afterwards, OGTT and HbA1c should be reassessed regularly to adjust the management as needed based on the latest glucose tolerance status. Patients with normal glycaemic measures (HbA1C <5.7% and FPG <5.6 mmol/l) for at least 1 year while receiving no active pharmacological therapy or ongoing procedures should be considered to be in complete diabetes remission (45, 46).

## Conclusions

Abnormal glucose metabolism is one of the most common complications of acromegaly and further contributes to an increased cardiovascular risk and mortality. Transsphenoidal surgery can notably improve glucose metabolism in patients with acromegaly. Decreased IR and β-cell functions and increased insulin sensitivity will be obtained in most patients after surgery regardless of their preoperative glucose tolerance status or whether they achieved acromegaly remission. Preoperative FCP >2.445 ng/ml is an excellent independent predictor of a postoperatively improved glucose tolerance status. OGTT and HbA1c should be reassessed regularly after surgery for acromegaly patients with abnormal glucose tolerance, and management should be adjusted as needed based on the patient's latest glucose tolerance status.

## Data Availability Statement

The datasets generated for this study are available on request to the corresponding author.

## Ethics Statement

All procedures involving human participants were performed in accordance with the ethical standards of the Institutional Ethics Committee of Peking Union Medical College Hospital at the Chinese Academy of Medical Sciences and Peking Union Medical College and with the 1964 Declaration of Helsinki and its later amendments or comparable ethical standards. Informed consent was obtained from all participants included in the study.

## Author Contributions

ZW wrote the main manuscript text. LG, XG, CF, KD, WL, MF, and XB collected, analyzed and interpreted the data. ZW and LG prepared figures and tables. BX designed the work, and critically revised it for important intellectual content. All authors reviewed the manuscript.

### Conflict of Interest

The authors declare that the research was conducted in the absence of any commercial or financial relationships that could be construed as a potential conflict of interest.
