# Supplementary material for: Preoperative Fasting C-Peptide Acts as a Promising Predictor of Improved Glucose Tolerance in Patients With Acromegaly After Transsphenoidal Surgery: A Retrospective Study of 64 Cases From a Large Pituitary Center in China
Source: Front Endocrinol (Lausanne). 2019 Nov 1;10:736. doi: 10.3389/fendo.2019.00736 (PMC6838023; doi:10.3389/fendo.2019.00736)
Supplement: Supplementary file 1 [file Table_1.DOCX]

**Supplementary Table 1:** Calculation formulas of indices of β-cell function, insulin sensitivity and insulin resistance.

1. Homeostasis assessment model of β-cell function (HOMA1-%β) = [FINS (μU/ml) ×20] / [FPG (mmol/l) -3.5]

2. Homeostasis assessment model of insulin sensitivity (HOMA1-%S) =22.5 / [FPG (mmol/l) × FINS (μU/ml)]

3. Homeostasis assessment model of insulin resistance (HOMA1-IR) = FPG (mmol/l) × FINS (μU/ml) / 22.5

4. Computer-based HOMA index of β-cell function (HOMA2-%β), insulin sensitivity (HOMA2-S%), and insulin resistance (HOMA2-IR) were calculated by the computer software downloaded from Diabetes Trials Unit (http://www.dtu.ox.ac.uk/homacalculator/download.php). HOMA2-%β (INS), HOMA2-%S (INS) and HOMA2-IR (INS) was calculated with FPG and FINS. HOMA2-%β (CP), HOMA2- %S (CP) and HOMA2-IR(CP) was calculated with FPG and FCP.

5. Insulin activity index (IAI) =1／[FPG (mmol/l) × FINS (μU/ml)]

6. Quantative insulin sensitivity check index (QUICKI) =1／[log FINS (μU/ml) + log FPG (mmol/l)]

7. AUC_PG_ (mmol/l) = (FPG+PG_30_) × 15 + (PG_30_+PG_60_) × 15 + (PG_60_+PG_120_) × 30 + (PG_120_+PG_180_) × 30.

AUC_INS_ (μU/ml) = (FINS+INS_30_) × 15 + (INS_30_+INS_60_) × 15 + (INS_60_+INS_120_) × 30 + (INS_120_+INS_180_) × 30.

AUC_CP_ (ng/ml) =(FCP+CP_30_) × 15 + (CP_30_+CP_60_) × 15 + (CP_60_+CP_120_) × 30 + (CP_120_+CP_180_) × 30.

8. Matsuda Index (whole body insulin sensitivity index, WBISI) =10000／{[FPG (mmol/l) × FINS (μU/ml)]^1/2^ × [PGmean × INSmean]^1/2^}.

PGmean (mmol/l) = (FPG+PG_30_+PG_60_+PG_120_+PG_180_) / 5.

INSmean (μU/ml) = (FINS+INS_30_+INS_60_+INS_120_+INS_180_) / 5.

9. Insulinogenic index (IGI) = (INS_30_-FINS) / (PG_30_-FPG) = △INS_30_ (μU/ml)/△PG_30_ (mmol/l).

10. Disposition index=IGI × Matsuda Index.

11. The OGTT insulin secretion sensitivity index 2 (ISSI2) = (AUC_INS_/ AUC_PG_) × Matsuda Index.

12. Modified β-cell function index (MBCI)＝[FPG (mmol/l) × FINS (μU/ml)] / [PG_120_ (mmol/l) + PG_60_ (mmol/l) - 7]

13. Estimated metabolic clearance rate of glucose (eMCR) = 19.240 - 0.281 × BMI (kg/m^2^) - 0.00498 × INS_120_ (μU/ml) - 0.333 × PG_120_ (mmol/l).

14. Estimated first phase insulin release (eFPIS) = 1283 + 1.829 × INS_30_ (μU/ml) - 138.7 × PG_30_ (mmol/l) + 3.772 × FINS (μU/ml).

15. Estimated second phase insulin release (eSPIS) = 286 + 0.416 × INS_30_ (μU/ml) - 25.94 × PG_30_ (mmol/l) + 0.926×FINS (μU/ml).
