# Supplementary material for: Preoperative Fasting C-Peptide Acts as a Promising Predictor of Improved Glucose Tolerance in Patients With Acromegaly After Transsphenoidal Surgery: A Retrospective Study of 64 Cases From a Large Pituitary Center in China
Source: Front Endocrinol (Lausanne). 2019 Nov 1;10:736. doi: 10.3389/fendo.2019.00736 (PMC6838023; doi:10.3389/fendo.2019.00736)
Supplement: Supplementary file 2 [file Table_2.DOCX]

Supplementary Table 2. Preoperative clinical characteristics of the diabetes mellitus (DM), prediabetes (PreDM) and normal glucose tolerance (NGT) group

| Parameters | DM (n=18) | PreDM (n=34) | NGT (n=12) | *P* value |
| --- | --- | --- | --- | --- |
| Age (yrs) | 42.8±10.9 | 41.0±10.0 | 39.0±14.2 | 0.659 |
| Sex (male:female) | 10:8 | 18:15 | 7:5 | 0.524 |
| Body mass index (kg/m^2^) | 27.7±4.4 | 25.3±3.2 | 25.5±4.5 | 0.110 |
| Hypertension (No. [%]) | 8(44.4%) | 9(26.5%) | 1(8.3%) | 0.261 |
| Disease duration (yrs) | 6.7±4.7 | 6.2±4.5 | 5.3±4.2 | 0.696 |
| TC | 4.8 (3.9-5.2) | 4.3 (3.8-4.8) | 3.9 (3.6-4.4) | 0.234 |
| TG | 1.5 (1.2-1.8) | 1.2 (1.0-1.5) | 1.2 (0.5-1.7) | 0.093 |
| Random GH (μg/L) | 15.0 (10.4-45.7) | 15.6 (8.9-35.6) | 14.1 (8.8-49.1) | 0.923 |
| Nadir GH (μg/L) | 13.0 (8.3-21.6) | 10.7 (5.1-31.2) | 12.9 (8.5-37.6) | 0.626 |
| IGF-1 (μg/L) | 922.0 (755.8-1091.5) | 859.5 (719.0-1025.5) | 899.0 (686.5-1022.5) | 0.539 |
| IGF-1 (%ULN) | 3.5±1.1 | 3.1±1.0 | 2.9±0.7 | 0.244 |
| HbA1c (%) | 7.1 (6.8-9.8) | 5.6 (5.5-5.7)* | 5.6 (5.4-5.7)* | **0.000** |
| FPG (mmol/L) | 7.3 (6.6-8.3) | 5.8 (5.3-6.3)* | 5.3 (5.1-5.4)* | **0.000** |
| 2h-PG (mmol/L) | 14.3 (11.5-15.9) | 8.9 (7.5-9.8)* | 5.7 (5.5-6.6)*^ | **0.000** |
| FINS (mU/L) | 15.3 (8.7-22.3) | 17.0 (12.7-23.1) | 18.2 (11.0-27.6)* | **0.048** |
| INS_120_ (mU/L) | 66.5 (33.6-92.0) | 97.2 (65.0-163.7)* | 81.4 (60.5-170.1) | **0.044** |
| FCP (ng/ml) | 1.8 (1.5-3.0) | 2.2 (1.8-3.2) | 2.5 (1.9-3.4)* | **0.048** |
| CP_120_ (ng/ml) | 5.4 (3.9-9.1) | 9.5 (6.9-11.8)* | 8.5 (6.6-10.9)* | **0.009** |
| Indices of β-cell function | | | | |
| HOMA1-%β (INS) | 90.0 (51.2-149.3) | 178.8 (102.3-209.3)* | 202.6 (131.3-340.5)* | **0.003** |
| HOMA2-%β (INS) | 79.5 (54.9-119.5) | 135.8 (94.5-154.1)* | 152.6 (113.0-215.7)* | **0.001** |
| HOMA2-%β (CP) | 56.9 (43.2-103.8) | 111.0 (86.7-138.7)* | 125.6 (110.0-159.2)* | **0.001** |
| AUC_PG_ | 2191.5 (1926.0-2529.4) | 1544.3 (1466.6-1683.8)* | 1190.3 (1122.4-1240.1)*^ | **0.000** |
| AUC_INS_ | 8588.3 (5405.5-15098.7) | 18758.5 (10128.2-27400.3)* | 17290.6 (13478.6-32335.1)* | **0.002** |
| AUC_CP_ | 796.4 (522.6-1304.4) | 1552.6 (1074.7-1759.7)* | 1325.3 (1214.2-1796.6)* | **0.002** |
| AUC_INS_/AUC_PG_ | 4.0 (1.9-7.6) | 12.7 (6.3-17.0)* | 14.7 (11.1-25.3)* | **0.000** |
| AUC_CP_/AUC_PG_ | 0.4 (0.2-0.7) | 1.0 (0.8-1.2)* | 1.2 (1.0-1.5)* | **0.000** |
| IGI | 0.3 (0.1-0.4) | 1.4 (0.6-2.0)* | 2.9 (1.8-3.8)* | **0.000** |
| IGI/IR | 0.04 (0.02-0.07) | 0.3 (0.2-0.5)* | 0.5 (0.4-0.8)* | **0.000** |
| Disposition Index (DI) | 0.5 (0.3-0.8) | 2.4 (1.5-3.4)* | 5.5 (3.1-6.8)*^ | **0.000** |
| ISSI2 | 8.2 (5.5-10.2) | 23.3 (18.2-28.6)* | 29.7 (27.9-38.5)*^ | **0.000** |
| MBCI | 7.4 (3.3-8.2) | 8.1 (5.5-12.2) | 14.3 (9.9-19.9)*^ | **0.004** |
| eFPIS (pmol/L) | 806.4 (214.2-1187.0) | 1757.4 (1168.3-2389.8)* | 3185.5 (1477.4-4321.4)* | **0.000** |
| eSPIS (pmol/L) | 234.1 (105.9-326.2) | 441.8 (327.8-605.8)* | 775.0 (377.8-1036.6)* | **0.000** |
| Indices of insulin sensitivity | | | | |
| HOMA1-%S (INS) | 16.3 (11.9-27.7) | 22.2 (16.3-31.7) | 23.3 (16.9-39.1) | 0.328 |
| HOMA2-%S (INS) | 37.4 (28.1-71.0) | 43.7(32.6-59.0) | 42.5 (30.1-69.8) | 0.891 |
| HOMA2-%S (CP) | 56.6 (40.7-75.2) | 52.1(43.1-70.2) | 62.2 (39.4-71.2) | 0.960 |
| QUICKI | 0.47 (0.44-0.52) | 0.50 (0.47-0.54) | 0.50 (0.47-0.57) | 0.328 |
| Matsuda Index (WBISI) | 1.8 (1.5-3.4) | 2.0 (1.5-2.6)* | 2.2 (1.3-3.3)* | **0.046** |
| eMCR (ml.kg^-1^.min^-1^) | 6.2 (5.3-7.1) | 8.8 (7.6-9.6)* | 9.8 (8.9-10.3)* | **0.000** |
| Indices of insulin resistance | | | | |
| HOMA1-IR (INS) | 6.2 (3.6-8.4) | 4.5 (3.2-6.1)* | 4.3 (2.6-6.2)* | **0.012** |
| HOMA2-IR (INS) | 2.7 (1.4-3.6) | 2.3 (1.7-3.1) | 2.4 (1.4-3.5) | 0.887 |
| HOMA2-IR (CP) | 1.8 (1.3-2.5) | 1.9 (1.4-2.3) | 1.6 (1.4-2.5) | 0.954 |
| IAI | 0.0072(0.0053-0.012) | 0.0099 (0.0073-0.014) | 0.010 (0.0075-0.017) | 0.328 |

TC total cholesterol, TG total triglycerides, GH growth hormone, IGF-1 insulin-like growth factor-1, ULN upper limit of normal, HbA1c glycosylated hemoglobin, FPG fasting plasma glucose, FINS fasting insulin, FCP fasting C-peptide, HOMA-%β homeostasis assessment model of β-cell function, AUC areas under the curve, IGI insulinogenic index, DI disposition index, ISSI2 the OGTT insulin secretion sensitivity index 2, MBCI modified β-cell function index, eFPIS estimated first phase insulin release, eSPIS estimated second phase insulin release, HOMA-%S homeostasis assessment model of insulin sensitivity, QUICKI quantative insulin sensitivity check index, WBISI whole body insulin sensitivity index, eMCR estimated metabolic clearance rate of glucose, HOMA-IR homeostasis assessment model of insulin resistance, IAI insulin activity index.

*P* values are for variations among the 3 groups; * means that *p* < 0.05 versus the DM group; ^ means that *p* < 0.05 versus the PreDM group.
