# Supplementary material for: Preoperative Fasting C-Peptide Acts as a Promising Predictor of Improved Glucose Tolerance in Patients With Acromegaly After Transsphenoidal Surgery: A Retrospective Study of 64 Cases From a Large Pituitary Center in China
Source: Front Endocrinol (Lausanne). 2019 Nov 1;10:736. doi: 10.3389/fendo.2019.00736 (PMC6838023; doi:10.3389/fendo.2019.00736)
Supplement: Supplementary file 5 [file Table_5.DOCX]

Supplementary Table 5. Comparisons of preoperative, immediately postoperative and 3-month postoperative parameters among the remission, GH/IGF-1 discordant and nonremission group

| Parameters | Remission Group (n=26) | | | | GH/IGF-1 Discordant Group (n=8) | | | | Nonremission Group (n=30) | | | |
| --- | --- | --- | --- | --- | --- | --- | --- | --- | --- | --- | --- | --- |
|  | Preop. | Immediately postop. | 3-month postop. | *P* value | Preop. | Immediately postop. | 3-month postop. | *P* value | Preop. | Immediately postop. | 3-month postop. | *P* value |
| Random GH (μg/L) | 10.6 (7.6-21.4) | 0.9 (0.7-1.5)* | 0.5 (0.2-0.7)* | **0.000** | 10.9 (8.1-18.9) | 1.6 (0.7-1.8)* | 1.6 (0.4-3.5)* | **0.002** | 26.5 (12.5-61.1) | 5.6 (3.0-15.4)* | 4.5 (2.5-9.1)* | **0.000** |
| Nadir GH (μg/L) | 7.5 (4.8-16.4) | 0.5 (0.3-0.6)* | 0.1 (0.06-0.3)*^ | **0.000** | 8.3 (6.4-10.9) | 0.7 (0.5-0.8)* | 0.3 (0.05-0.4)*^ | **0.001** | 17.1 (9.8-37.1) | 4.0 (1.8-11.0)* | 2.1 (1.3-6.1)*^ | **0.000** |
| IGF-1 (μg/L) | 875.5 (714.5-1015.8) | 503.0 (428.5-669.3)* | 231.0 (198.5-270.8)*^ | **0.000** | 921.5 (795.8-1123.5) | 616.5 (571.5-714.5)* | 382.0 (300.1-459.3)*^ | **0.000** | 910.5 (720.5-1071.3) | 811.0 (585.3-931.3)* | 604.5 (513.8-761.0)*^ | **0.000** |
| IGF-1 (%ULN) | 3.2±0.9 | 2.1±0.9* | 0.9±0.1*^ | **0.000** | 3.2±0.8 | 2.3±0.7* | 1.3±0.4*^ | **0.000** | 3.2±1.2 | 2.8±1.1* | 2.2±0.7*^ | **0.000** |
| HbA1c (%) | 5.7 (5.6-6.2) | 5.6 (5.4-6.0)* | 5.3 (5.1-5.4)*^ | **0.000** | 5.5 (5.5-5.6) | 5.4 (5.4-5.5) | 5.1 (5.0-5.2)*^ | **0.001** | 5.8 (5.7-7.0) | 5.7 (5.6-6.8)* | 5.5 (5.3-5.9)*^ | **0.000** |
| FPG (mmol/L) | 5.8 (5.2-6.6) | 5.6 (5.2-5.9)* | 5.2 (4.8-5.4)*^ | **0.001** | 5.4 (5.2-5.6) | 5.8 (5.3-6.2) | 5.2 (4.9-5.4) | 0.250 | 6.2 (5.4-6.9) | 6.7 (5.8-7.5) | 5.5 (5.3-6.2)*^ | **0.000** |
| 2h-PG (mmol/L) | 8.9 (7.0-10.8) | 8.5 (6.7-9.9) | 5.1 (4.4-6.6)*^ | **0.000** | 6.9 (6.4-8.7) | 8.4 (7.7-9.8) | 5.4 (5.0-6.2)*^ | **0.010** | 9.7 (7.9-13.4) | 12.4 (8.8-15.2) | 6.8 (5.6-9.4)*^ | **0.000** |
| FINS (mU/L) | 16.3 (10.1-21.1) | 7.9 (6.0-11.0)* | 6.0 (4.2-7.6)*^ | **0.000** | 12.6 (7.6-20.9) | 10.1 (8.7-15.8) | 9.1 (7.1-10.9) | 0.093 | 23.6 (13.3-28.0) | 15.6 (11.0-28.5) | 10.7 (7.6-15.4)*^ | **0.000** |
| INS_120_ (mU/L) | 72.6 (60.3-94.3) | 52.6 (31.4-95.7) | 20.4 (11.7-33.1)*^ | **0.000** | 92.2 (70.3-120.8) | 88.7 (56.4-102.6) | 61.9 (47.7-78.2)* | 0.072 | 94.7 (55.1-163.3) | 95.7 (60.9-178.0) | 53.6 (35.0-69.0)*^ | **0.001** |
| FCP (ng/ml) | 2.0 (1.8-2.6) | 1.5 (1.3-1.8)* | 1.2 (1.0-1.3)* | **0.000** | 1.8 (1.5-2.6) | 2.1 (1.5-2.3) | 1.4 (1.3-1.5)*^ | **0.043** | 2.9 (1.9-3.5) | 2.7 (2.0-3.6) | 1.9 (1.5-3.2)*^ | **0.007** |
| CP_120_ (ng/ml) | 8.3 (6.1-9.3) | 8.9 (5.3-11.6)* | 4.5 (3.7-6.7)* | **0.000** | 8.6 (6.3-10.1) | 10.0 (7.9-12.8) | 7.4 (6.2-8.7)*^ | **0.042** | 9.5 (5.0-11.1) | 11.2 (7.9-14.2)* | 7.1 (5.2-8.0)*^ | **0.001** |
| Indices of β-cell function | | | | | | | | | | | | |
| HOMA1-%β (INS) | 119.6 (88.7-193.7) | 83.5 (48.8-111.6) | 68.3 (52.1-107.0)* | **0.006** | 144.4 (77.1-221.8) | 94.6 (88.5-131.5) | 101.3 (81.9-138.8) | 0.197 | 169.8 (122.1-220.9) | 112.9 (60.7-201.3)* | 107.5 (64.3-150.3)* | **0.001** |
| HOMA2-%β (INS) | 102.9 (78.2-147.5) | 82.7 (56.7-101.4)* | 72.7 (60.3-94.8)* | **0.010** | 119.8 (78.9-162.3) | 88.9 (81.8-110.3) | 94.7 (80.3-114.3) | 0.197 | 130.0 (99.9-160.8) | 100.3 (61.7-141)* | 98.8 (69.4-122.7)* | **0.001** |
| HOMA2-%β (CP) | 102.7 (60.0-126.6) | 86.2 (67.9-102.7) | 81.7 (68.8-100.9) | 0.135 | 106.6 (84.0-140.8) | 90.9 (74.2-106.4) | 90.2 (81.9-107.1) | 0.417 | 113.0 (75.3-137.5) | 101.1 (67.3-124.2) | 101.3 (79.3-117.6) | 0.107 |
| AUC_PG_ | 1546.5 (1403.6-1785.4) | 1432.5 (1290.0-1608.4) | 1136.3 (1037.3-1374.8)*^ | **0.000** | 1281.0 (1219.5-1560.4) | 1498.5 (1368.8-1696.1) | 1091.3 (1014.8-1228.1)*^ | **0.001** | 1686.8 (1497.8-2143.2) | 1923.0 (1560.8-2338.9) | 1318.5 (1165.5-1640.6)*^ | **0.000** |
| AUC_INS_ | 12475.8 (9087.0-20581.0) | 9057.2 (6889.7-14605.9)* | 5341.7 (3645.0-7863.6)*^ | **0.000** | 18538.7 (13333.6-21244.3) | 16757.5 (12047.1-19628.3) | 10610.7 (8531.2-11554.2 )* | 0.093 | 16871.6 (8129.1-25529.6) | 19679.9 (9452.7-29122.3) | 10298.3 (5939.4-14253.8)*^ | **0.000** |
| AUC_CP_ | 1160.0 (889.9-1665.1) | 1354.5 (792.1-1459.4) | 719.3 (643.7-1074.6)*^ | **0.000** | 1323.4 (1062.9-1604.6) | 1465.8 (1249.7-1855.7) | 1116.5 (962.3-1330.5)*^ | **0.005** | 1432.0 (787.3-1795.4) | 1875.2 (1170.2-2134.5)* | 1130.9 (836.6-1358.3)*^ | **0.000** |
| AUC_INS_/AUC_PG_ | 7.9 (5.6-15.3) | 6.0 (2.9-11.2)* | 4.3 (3.2-7.1)* | **0.000** | 14.7 (11.3-15.6) | 12.7 (7.7-13.9) | 9.2 (8.7-9.6)* | 0.093 | 9.2 (5.0-15.8) | 9.9 (5.0-16.8) | 7.7 (4.4-10.8)*^ | **0.024** |
| AUC_CP_/AUC_PG_ | 0.8 (0.5-1.2) | 0.9 (0.5-1.1) | 0.7 (0.6-0.8) | 0.089 | 1.1 (0.8-1.2) | 1.1 (0.9-1.2) | 1.1 (0.9-1.2) | 0.687 | 0.8 (0.4-1.2) | 1.0 (0.5-1.2) | 0.9 (0.6-1.0) | 0.531 |
| IGI | 0.7 (0.4-2.0) | 0.5 (0.3-1.5) | 0.6 (0.3-0.9) | 0.060 | 1.6 (1.2-2.5) | 1.0 (0.7-2.2) | 1.3 (0.8-2.0) | 0.687 | 0.9 (0.4-1.8) | 1.2 (0.4-2.3) | 0.7 (0.3-1.6)* | 0.648 |
| IGI/IR | 0.2 (0.1-0.5) | 0.3 (0.2-0.4) | 0.4 (0.2-0.6)* | **0.001** | 0.4 (0.3-0.5) | 0.3 (0.1-0.8) | 0.6 (0.4-1.0) | 0.197 | 0.2 (0.04-0.3) | 0.2 (0.1-0.5) | 0.3 (0.1-0.6)* | **0.032** |
| Disposition Index | 1.8 (1.4-3.5) | 2.5 (1.6-3.6) | 3.6 (2.3-6.5)*^ | **0.011** | 3.4 (2.6-4.3) | 2.5 (1.4-5.2) | 5.4 (3.6-7.4)*^ | **0.010** | 1.5 (0.5-2.8) | 1.7 (0.9-3.6) | 2.5 (1.4-4.6)*^ | **0.001** |
| ISSI2 | 25.2 (14.1-28.4) | 24.9 (21.1-32.0)* | 33.0 (25.0-39.5)*^ | **0.000** | 31.1 (24.9-32.6) | 27.4 (16.6-36.3) | 39.5 (35.7-45.2)*^ | **0.002** | 18.5 (8.9-25.5) | 17.8 (9.0-25.1) | 26.2 (17.9-34.4)*^ | **0.000** |
| MBCI | 7.7 (5.2-10.0) | 4.6 (2.4-5.8)* | 5.0 (2.7-7.8)* | **0.014** | 8.6 (4.3-14.1) | 6.3 (4.3-6.6) | 9.1 (5.6-10.1) | 0.093 | 10.6 (6.3-12.9) | 7.4 (4.6-11.0) | 7.1 (4.0-10.7) | 0.061 |
| eFPIS (pmol/L) | 1235.6 (787.5-2318.8) | 852.3 (440.7-1529.7)* | 849.8 (516.0-1082.9)* | **0.028** | 1823.7 (1437.3-2398.3) | 1386.3 (1037.1-1805.9)* | 1233.7 (1034.9-1517.4)* | **0.042** | 1539.8 (1067.9-2537.0) | 1443.6 (622.9-2811.5) | 1085.3 (693.8-1762.3)*^ | **0.003** |
| eSPIS (pmol/L) | 340.3 (236.0-569.9) | 245.0 (148.9-395.1)* | 237.7 (169.8-284.6)* | **0.006** | 462.1 (370.1-596.2) | 371.6 (284.4-456.0)* | 315.8 (281.2-383.2)* | **0.047** | 403.0 (311.2-641.9) | 384.2 (196.4-690.8) | 294.7 (204.9-456.0)*^ | **0.002** |
| Indices of insulin sensitivity | | | | | | | | | | | | |
| HOMA1-%S (INS) | 24.3 (18.6-39.5) | 51.5 (37.7-58.3)* | 69.9 (53.1-99.9)*^ | **0.000** | 33.7 (20.6-49.3) | 38.0 (27.0-45.9) | 51.6 (41.8-62.1)*^ | **0.030** | 17.4 (11.6-26.6) | 22.1 (10.8-31.7) | 38.0 (25.2-49.7)*^ | **0.000** |
| HOMA2-%S (INS) | 47.4 (37.0-75.1) | 96.2 (68.7-122.4)* | 125.3 (100.1-182.4)*^ | **0.000** | 60.7 (38.0-97.5) | 73.4 (48.2-88.0) | 85.5 (71.5-107.3)*^ | **0.042** | 32.7 (27.0-55.7) | 42.1 (27.3-65.8) | 70.3 (49.2-94.5)*^ | **0.000** |
| HOMA2-%S (CP) | 59.7 (49.8-72.9) | 89.8 (72.3-102.0)* | 117.5 (101.2-132.5)*^ | **0.000** | 72.9 (52.7-87.4) | 60.9 (58.3-91.0) | 96.0 (89.8-103.0)*^ | **0.043** | 48.0 (37.0-64.5) | 48.1 (35.1-63.9) | 71.5 (60.0-87.9)*^ | **0.001** |
| QUICKI | 0.51 (0.48-0.57) | 0.61 ((0.56-0.63)* | 0.66 (0.61-0.74)*^ | **0.000** | 0.55 (0.49-0.60) | 0.56 (0.52-0.59) | 0.61 (0.57-0.64)*^ | **0.030** | 0.47 (0.44-0.52) | 0.50 (0.43-0.54) | 0.56 (0.50-0.60)*^ | **0.000** |
| Matsuda Index (WBISI) | 2.3 (1.7-3.5) | 4.3 (2.9-5.4)* | 7.1 (5.4-8.3)* | **0.000** | 2.2 (1.9-4.0) | 2.8 (2.3-3.3) | 4.5 (3.8-4.9)*^ | **0.010** | 1.7 (1.3-2.5) | 1.9 (1.4-2.6) | 3.4 (2.6-4.9)*^ | **0.000** |
| eMCR (ml/kg/min) | 8.8 (7.4-9.6) | 9.0 (8.1-9.5) | 10.0 (9.3-10.8)*^ | **0.000** | 9.5 (8.5-10.1) | 8.7 (7.9-9.8) | 10.3 (9.9-10.5)*^ | **0.010** | 7.5 (6.2-9.0) | 7.1 (5.8-8.4) | 9.2 (7.7-9.7)*^ | **0.000** |
| Indices of insulin resistance | | | | | | | | | | | | |
| HOMA1-IR (INS) | 4.1 (2.5-5.4) | 1.9 (1.7-2.7)* | 1.4 (1.0-1.9)*^ | **0.000** | 3.0 (2.0-5.0) | 2.6 (2.2-3.7) | 2.0 (1.6-2.4)*^ | **0.030** | 5.8 (3.8-8.6) | 4.5 (3.2-9.2) | 2.6 (2.0-4.0)*^ | **0.000** |
| HOMA2-IR (INS) | 2.1 (1.3-2.7) | 1.0 (0.8-1.5)* | 0.8 (0.6-1.0)*^ | **0.000** | 1.7 (1.0-2.7) | 1.4 (1.1-2.1) | 1.2 (0.9-1.4)*^ | **0.042** | 3.1 (1.8-3.7) | 2.4 (1.5-3.7) | 1.4 (1.1-2.0)*^ | **0.000** |
| HOMA2-IR (CP) | 1.7 (1.4-2.0) | 1.1 (1.0-1.4)* | 0.9 (0.8-1.0)*^ | **0.000** | 1.4 (1.2-1.9) | 1.6 (1.1-1.7) | 1.0 (1.0-1.1)*^ | **0.043** | 2.1 (1.6-2.7) | 2.1 (1.6-2.9) | 1.4 (1.1-1.7)*^ | **0.000** |
| IAI | 0.011 (0.008-0.018) | 0.023 (0.017-0.026)* | 0.031 (0.024-0.044)*^ | **0.000** | 0.015 (0.009-0.022) | 0.017 (0.012-0.020) | 0.023 (0.019-0.028)*^ | **0.030** | 0.008 (0.005-0.012) | 0.0098 (0.0048-0.014) | 0.017 (0.011-0.021)*^ | **0.000** |

TC total cholesterol, TG total triglycerides, GH growth hormone, IGF-1 insulin-like growth factor-1, ULN upper limit of normal, HbA1c glycosylated hemoglobin, FPG fasting plasma glucose, FINS fasting insulin, FCP fasting C-peptide, HOMA-%β homeostasis assessment model of β-cell function, AUC areas under the curve, IGI insulinogenic index, DI disposition index, ISSI2 the OGTT insulin secretion sensitivity index 2, MBCI modified β-cell function index, eFPIS estimated first phase insulin release, eSPIS estimated second phase insulin release, HOMA-%S homeostasis assessment model of insulin sensitivity, QUICKI quantative insulin sensitivity check index, WBISI whole body insulin sensitivity index, eMCR estimated metabolic clearance rate of glucose, HOMA-IR homeostasis assessment model of insulin resistance, IAI insulin activity index.

*P* values are for variations among the preoperative, immediately postoperative and 3-month postoperative groups; * means that *p* < 0.05 versus the preoperative group; ^ means that *p* < 0.05 for immediately postoperative group versus 3-month postoperative group.
